# Intimate Partner Violence (IPV) and Associated Factors in HPTN 071 (PopART) Study Communities in Zambia and South Africa—A Comparison by HIV Status

**DOI:** 10.1007/s10461-021-03492-6

**Published:** 2022-02-14

**Authors:** K. Sabapathy, H. Stöckl, C. Mulubwa, C. Mubekapi-Musadaidzwa, G. Hoddinott, S. Floyd, J. Seeley, V. Bond, P. Bock, S. Fidler, H. Ayles, R. Hayes, on behalf of HPTN 071 (PopART) Study Team

**Affiliations:** 1 London School of Hygiene and Tropical Medicine, Keppel Street, London, UK; 2 Ludwig-Maximilians-University, Munich, Germany; 3 Zambia AIDS Related TB Project, University of Zambia, Lusaka, Zambia; 4 Desmond Tutu TB Centre, Stellenbosch University, Stellenbosch, Western Cape South Africa; 5 Imperial College London, London, UK

**Keywords:** Intimate Partner Violence, HIV, Universal testing and treatment, Sub-Saharan Africa

## Abstract

The HPTN 071(PopART) study was a community-randomised trial in Zambia and South Africa, examining the impact of combination-prevention including universal testing and treatment (UTT), on HIV-incidence. This sub-study evaluated factors associated with IPV (physical and/or sexual) to identify differences by HIV status. During 2015–16, a random subset of adults who participated in the first year of the PopART intervention were recruited and standardised questionnaires were administered. Logistic regression was performed to estimate odds ratios of factors associated with IPV. Among > 700 women studied (300 HIV-negative;400 HIV-positive), ~ 20% reported experiencing physical and/or sexual violence in the last 12-months. Sexual violence was similar by HIV status, but physical violence and reporting both physical/sexual violence was more common among HIV-positive women. Spending nights away from the community in the last 12-months was associated with higher odds of IPV among both HIV-negative (aOR 3.17, 95% CI 1.02–9.81) and HIV-positive women (aOR 1.79, 95% CI 0.99–3.24). Among HIV-positive women, financial autonomy was associated with reduced IPV (aOR:0.41,95%CI:0.23-0.75) while pregnancy in the last 12-months (aOR 2.25, 95% CI 1.07–4.74), risk of alcohol dependence (aOR 2.75, 95% CI 1.51–5.00) and risk of mental distress (aOR 2.62, 95% CI 1.33–5.16) were associated with increased IPV. Among HIV-negative women reporting sex in the last 12-months, transactional sex (aOR 3.97, 95% CI 1.02–15.37) and not knowing partner’s HIV status (aOR 3.01, 95% CI 1.24–7.29) were associated with IPV. IPV was commonly reported in the study population and factors associated with IPV differed by HIV status. The association of mobility with IPV warrants further research. The high prevalence of harmful alcohol use and mental distress, and their association with IPV among HIV-positive women require urgent attention.

## Resumen

El estudio HPTN 071 (PopART) fue un ensayo aleatorio-comunitario realizado en Zambia y Sudáfrica, que examinó el impacto de la prevención combinada, incluyendo las pruebas y tratamiento universal (UTT), en la incidencia del VIH. Este subestudio evaluó los factores asociados con la IPV (físicos y / o sexuales) para identificar diferencias en el estado del VIH. Durante 2015-16, un subconjunto aleatorio de adultos fueron reclutados para participar en el primer año de intervención de PopART, donde se administraron cuestionarios estandarizados. Se realizó una regresión logística para estimar las ratios de probabilidad de los factores asociados con la VPI. Entre las > 700 mujeres estudiadas (300 VIH negativas; 400 VIH positivas), ~ 20% informó haber experimentado violencia física y / o sexual en los últimos 12 meses. La violencia sexual fue similar en cuanto al estado del VIH. La denuncia de violencia física y sexual fue más común entre las mujeres VIH positivas. Pasar noches fuera de la comunidad en los últimos 12 meses, se asoció con mayores probabilidades de VPI entre las mujeres VIH negativas (ORa 3,17, 95% IC 1,02–9,81) y las mujeres VIH positivas (ORa 1,79, 95% IC 0,99–3,24). Entre las mujeres VIH positivas, la autonomía financiera se asoció con una reducción de la VPI (ORa 0,41; IC del 95% 0,23-0,75) mientras que en el embarazo en los últimos 12 meses (ORa 2,25; IC del 95% 1,07–4,74), riesgo a la dependencia del alcohol (ORa 2,75% IC 1,51–5,00) y el riesgo de angustia mental (ORa 2,62% IC del 95% 1,33–5,16) se asociaron con un aumento de la VPI. Entre las mujeres VIH negativas que informaron haber tenido relaciones sexuales en los últimos 12 meses, el sexo transaccional (ORa 3.97, 95% CI 1.02–15.37) y el desconocimiento del estado de VIH de la pareja (ORa 3.01, 95% CI 1.24–7.29) se asociaron con IPV. La IPV fue notificada mayoritariamente en la población de estudio y los factores asociados con la IPV diferían según el estado del VIH. La asociación de la movilidad con la IPV justifica una mayor investigación. La alta prevalencia de l consumo nocivo de alcohol y la angustia mental, y su asociación con la VPI entre las mujeres seropositivas, requieren atención urgente.

## Introduction

Gender-based violence, and especially intimate partner violence (IPV), are widespread [1]. At least one in three women worldwide report IPV or non-partner sexual violence throughout their life-time. Sub-Saharan Africa has among the highest regional estimates with 37% of ever-partnered women reporting lifetime IPV and in Zambia this was as high as 43% in a demographic and health survey (DHS) in 2015 [1, 2]. A clear association between physical and/or sexual violence and HIV has been shown in sub-Saharan Africa [3]. It is thought to be driven by relationship power imbalances and gender inequality that leaves women with limited room to negotiate safe sex in relationships [4]. There is evidence that IPV or the fear of IPV impacts on women’s and girls’ ability to access services and lowers HIV treatment adherence and therefore increases HIV progression [5]. Disclosure of HIV positive status risks a violent reaction from partners which can increase isolation, restrict access to social support networks that aid adherence, or may increase depression and anxiety that lead to missed medication (intentionally or accidentally) [4–7]. While there is a strong body of evidence on IPV being associated with HIV infection, young age, alcohol abuse, mental health issues and economic instability, there is a gap in knowledge on whether these factors affect the occurrence of IPV similarly in HIV positive and HIV negative women [8–10].

The HPTN 071 [Population effects of Antiretroviral therapy to Reduce HIV Transmission (PopART)] trial was conducted in 21 urban/peri-urban communities in Zambia and South Africa (2013–2018) to examine the impact of a combination prevention package including universal testing and treatment (UTT) on HIV incidence at a community level. The PopART intervention achieved a 20–30% reduction in HIV incidence [11].

We conducted a cross-sectional analysis of factors associated with IPV among women living in PopART intervention communities during the trial intervention period, to explore factors associated with IPV and identify differences by HIV status.

## Methods

**Fig. 1:**
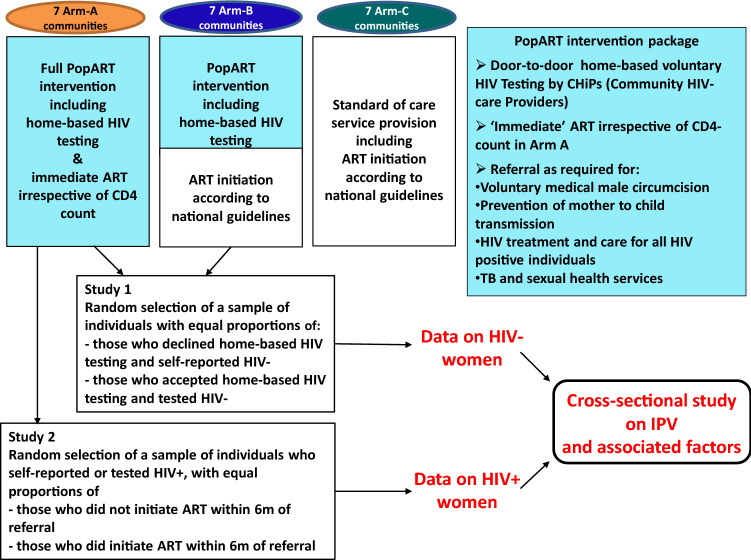
Overview of HPTN 071 (PopART) trial during the first year of the intervention and sampling frame of current study

The HPTN 071 (PopART) trial consisted of three trial arms as described elsewhere (Fig. 1) [12]. In trial Arms A and B, home-based HIV testing services (HB-HTS) were offered to all community members. In Arm A, treatment irrespective of CD4-criteria was offered to all people living with HIV as an intervention from the beginning of the trial, prior to incorporation into World Health Organization (WHO) or national guidelines. During 2015, approximately a year into delivery of the trial intervention, two nested research studies were conducted to examine the acceptability of the PopART “universal testing and treatment” interventions. The first examined factors associated with the uptake of the PopART home-based “universal testing” intervention which was delivered in Arm-A and Arm-B communities. A random subset of adults (≥ 18 years) from each community who accepted HB-HTS (controls) and an equal number who declined HB-HTS (cases) were recruited [13]. The second study examined uptake of the “universal treatment” intervention which meant all PLHIV (irrespective of CD4-count) were eligible for ART. This was only available in Arm A communities during the first year of the trial. A random subset of PLHIV who successfully initiated ART within six months of referral (controls) and an equal number who delayed or did not start ART (cases) were recruited for the second study [14].

Inclusion into the current analysis on IPV involved all women from the first study who tested HIV negative with PopART community health workers or self-reported HIV negative status, and all women living with HIV from the second study (Fig. 1). As such, participants in this study were randomly selected from the PopART intervention communities during the first year of the trial, stratified by acceptance/refusal of HB-HTS (HIV negative participants) and initiation/non-initiation of timely ART (HIV positive participants).

Demographic, socio-economic, behavioural factors and characteristics related to participants’ health and HIV status were surveyed using English language standardised questionnaires administered by research assistants fluent in English and the local vernacular. Research assistants received extensive training to ensure standardised use of terminology if they had to translate questions. Pilot testing of the questionnaires was also done to enhance consistency. All women irrespective of relationship status at time of survey were asked about experience of IPV using questions based on the DHS module on domestic violence [15]: “In the last 12 months, how often has a partner physically hurt you e.g. slapped, kicked, pushed, punched, beaten or otherwise physically hurt you?” and “In the last 12 months, how often has a partner made you have sexual activities when you did not want to?”. Any woman who provided an affirmative response (once, a few times or often) to one or both questions was considered to have experienced IPV (either sexual, physical or both sexual and physical). Verbal abuse was also measured with the question “In the last 12 months, how often has a partner verbally insulted you or humiliated you in front of other people, or intimidated or threatened to hurt you?”.

Logistic regression was performed to estimate odds ratios (ORs) and all crude models included community, case/control status of the original study and age category as a priori potentially confounding variables. Additional variables which are known to be associated with IPV and HIV [16] and which showed evidence of association with IPV (p < 0.05) were included in the multivariable models. If co-linearity was plausible and supported by cross-tabulation of the data (e.g., being head of the household and having control of household finances), only the variable which was more strongly associated with IPV was retained in the multi-variable models.

The data on HIV negative and HIV positive women were drawn from two different studies (as described above) and two separate models were run accordingly. When data were only relevant for a subset of participants, the model automatically excluded individuals on whom there were no data (e.g. in relation to sexual behaviour in the previous 12 months, individuals who reported no sexual activity in the previous 12 months were excluded from the outset). Likelihood ratio testing (LRT) was done to assess the statistical evidence of association. For variables with three or more response categories and plausible rationale for a dose–response relationship, tests for trend were performed.

The study was approved by the ethics committees of the University of Zambia, Stellenbosch University and London School of Hygiene and Tropical Medicine.

## Results

**Fig. 2:**
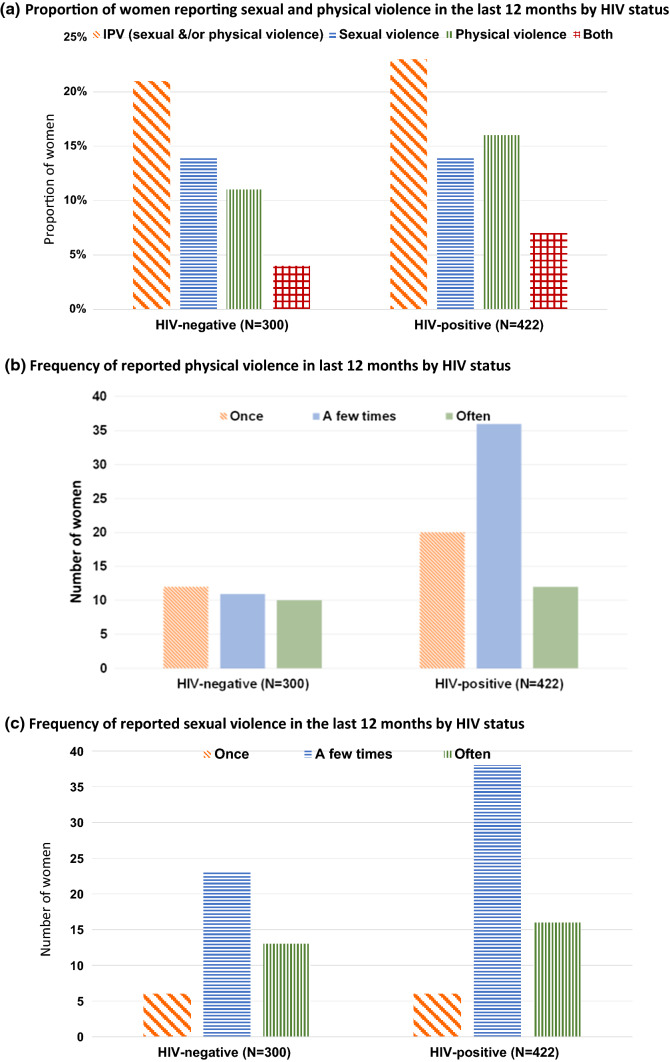

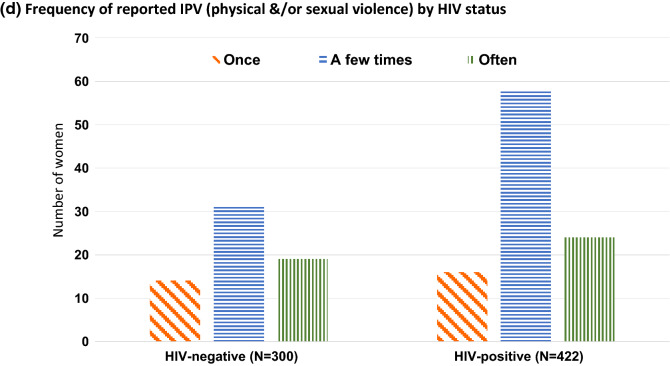
**a** Proportion of women reporting sexual and physical violence in the last 12 months by HIV status. **b** Frequency of reported physical violence in last 12 months by HIV status. **c** Frequency of reported sexual violence in the last 12 months by HIV status. **d** Frequency of reported IPV (physical &/or sexual violence) by HIV status

The analysis included 300 HIV negative women and 422 HIV positive women (median age 31 years (IQR 23–40) and 34 years (IQR 28–42), respectively). Among 722 women studied, ~ 20% of women reported at least one episode of physical and/or sexual violence in the last 12 months [64/300 (21.3%) HIV negative and 98/422 (23.2%) HIV positive] (Fig. 2a). Figure 2b–d illustrate that the majority of those who reported IPV experienced it more than once in the last 12 months with 29.7% (19/64) of HIV negative women and 24.5% (24/98) of HIV positive women who reported IPV saying they experienced it often and a further 48.4% (31/64) and 59.2% (58/98) respectively, reporting a few episodes of IPV in the last 12 months. The proportion of women reporting sexual violence was similar by HIV status (~ 14%), but HIV positive women were more likely to report physical violence (16.1% vs. 11.0%) and both sexual and physical violence (7.1% vs. 3.6%), compared to HIV negative women. Thirty-nine percent (n = 25) of HIV negative and 51.0% (n = 50) of HIV positive women who experienced IPV also reported verbal abuse.

### Characteristics of Women in the Study

The HIV negative women studied included 55.0% (n = 165) who had accepted HB-HTS and 45.0% (n = 135) who had declined (Table 1). Most women were married and unemployed. The majority of HIV negative women had secondary education or higher (68.3%, n = 205) while most HIV positive women had primary education (52.4%, n = 221). A greater proportion of HIV positive women were the head of their household (35.3%, n = 149) compared to HIV negative women (26.3%, n = 79) and reported autonomy over household finances [39.6% (n = 167) vs 30.0%, n = 90]. Most women had spent at least one night away from the community in the last 12 months. Higher proportions of HIV positive women reported more sexual partners compared to HIV negative women, for example 23.9%, (n = 101) reported 5 or more lifetime partners while only 8.3% (n = 25) of HIV negative women reported the same. Harmful alcohol use was relatively high with 16.0% (n = 48) of HIV negative women and 19.7% (n = 83) of HIV positive women meeting the criteria which suggest risk of alcohol dependency (score ≥ 8) on the WHO alcohol use and disorders identification test (AUDIT) [17]. Prevalence of mental distress among women studied was also high [HIV negative 18.3%, N = 55 and HIV positive 14.9% (n = 63)], as measured by the modified WHO 10-scale weighted self-reported questionnaire (SRQ-10) [18].

**Table 1:** IPV and associated factors, by HIV-status

	HIV- [N = 300, median age:31y (IQR:23–40)]					HIV + [N = 422, median age 34y (IQR:28–42)]				
	IPV n/N (%)	OR^a^	95% CI	aOR^a^	95% CI	IPV n/N (%)	OR^a^	95% CI	aOR^a^	95% CI
Accepted HB-HTS	34/165 (20.6)	1	*p* = *0.37*	1	*p* = *0.65*					
Declined HB-HTS	30/135 (22.2)	1.32	0.72–2.43	1.18	0.57–2.50					
Started ART within 6 m						53/221 (24.0)	1	*p* = *0.59*	1	*p* = *0.78*
Did not start ART within 6 m						45/201 (22.4)	0.88	0.55–1.40	0.81	0.48–1.37
*Demographic characteristics*										
Age category										
18–24y	29/97 (29.9)	1	*p* = *0.07*	1	*p* = *0.31*	17/60 (28.3)	1	*p* = *0.03*	1	*p* = *0.46*
25–34y	20/84 (23.8)	0.93	0.46–1.91	0.90	0.34–2.42	42/161 (26.1)	0.87	0.45–1.72	0.93	0.44–1.99
35—44y	9/62 (14.5)	0.48	0.20–1.17	0.52	0.16–1.70	28/113 (24.8)	0.84	0.41–1.73	1.14	0.50–2.59
45years and older	6/57 (10.5)	0.34	0.12–0.92	0.36	0.10–1.26	11/88 (12.5)	0.33	0.14–0.79	0.59	0.22–1.55
Marital status										
Currently married	48/174 (27.6)	1	*p* < *0.001*	1	*p* = *0.07*	57/224 (25.4)	1	*p* = *0.21*	1	*p* = *0.20*
Never married	7/74 (9.5)	0.17	0.06–0.47	0.26	0.08–0.87	14/66 (21.2)	0.51	0.24–1.10	0.19	0.21–1.36
Previously married	9/52 (17.3)	0.65	0.27–1.57	0.75	0.24–2.28	27/132 (20.5)	0.87	0.50–1.50	2.11	1.01–4.39
*Socioeconomic factors*										
Educational status										
Primary	22/95 (23.2)	1	*p* = *0.97*	1	*p* = *0.08*	49/221 (22.2)	1	*p* = *0.56*	1	*p* = *0.51*
Secondary or above	42/205 (20.5)	0.99	0.48–2.04	2.33	0.90–6.03	49/200 (24.5)	0.85	0.50–1.45	0.83	0.47–1.46
Employment status										
Unemployed	50/201 (24.9)	1	*p* = *0.06*	1	*p* = *0.07*	68/271 (25.1)	1	*p* = *0.22*	1	*p* = *0.59*
Employed	14/99 (14.1)	0.48	0.23–1.01	0.43	0.17–1.07	30/151 (19.9)	0.73	0.43–1.21	1.17	0.66–1.09
Participant is head of HH										
N	52/221 (23.5)	1	*p* = *0.43*	1	*p* = *0.44*	77/273 (28.2)	1	*p* = *0.01*	1	*p* = *0.93*
Y	12/79 (15.2)	0.72	0.31–1.63	1.62	0.47–5.56	21/149 (14.1)	0.49	0.27–0.86	1.03	0.48–1.22
Control of household finances										
N	54/210 (25.7)	1	*p* = *0.03*	1	*p* = *0.10*	77/255 (30.2)	1	*p* < *0.001*	1	*p* = *0.004*
Y	10/90 (11.1)	0.40	0.17–0.93	0.44	0.16–1.18	21/167 (12.6)	0.37	0.21–0.64	0.41	0.23–0.75
*Sexual risk behaviour, HIV and other health related factors*										
Night(s) away from community in last 12 m										
N	6/69 (8.7)	1	*p* = *0.04*	1	*p* = *0.05*	25/153 (16.3)	1	*p* = *0.02*	1	*p* = *0.05*
Y	45/182 (24.7)	2.59	0.97–6.91	3.17	1.02–9.81	71/263 (27.0)	1.95	1.13–3.39	1.79	0.99–3.24
Lifetime no. of partners										
1–2	36/179 (20.1)	1	*p* = *0.006*	1	*p* = *0.09*	32/165 (19.4)	1	*p* = *0.50*	1	*p* = *0.59*
≥ 3 (HIV-ve) 3–4 (HIV + ve)	27/101 (26.7)	2.67	1.33–5.34	2.06	0.89—4.77	41/155 (26.5)	1.39	0.79–2.45	1.37	0.74–2.54
≥ 5 (HIV + ve)	b	b	b	b	b	25/101 (24.8)	1.28	0.67–2.44	1.14	0.55–2.37
Pregnant in the last 12 m										
N	50/253 (19.8)	1	*p* = *0.74*	1	*p* = *0.32*	80/378 (21.2)	1	*p* = *0.02*	1	*p* = *0.03*
Y	14/47 (29.8)	1.14	0.52–2.50	0.58	0.20–1.71	18/44 (40.9)	2.32	1.18–4.60	2.25	1.07–4.74
Risk of alcohol dependence (AUDIT score)										
N (≤ 7/10)	53/277 (19.1)	1	*p* = *0.04*	1	*p* = *0.15*	65/339 (19.2)	1	*p* = *0.001*	1	*p* = *0.02*
Y (≥ 8/10)	11/23 (47.8)	2.75	1.07–7.02	2.88	0.85–9.75	33/83 (39.8)	2.55	1.48–4.39	2.75	1.51–5.00
Risk of mental distress (SRQ-10 weighted score)										
N (≤ 6/20)	46/245 (18.8)	1	*p* = *0.10*	1	*p* = *0.45*	73/359 (20.3)	1	*p* = *0.001*	1	*p* = *0.005*
Y (≥ 7/20)	18/55 (32.7)	1.90	0.88–4.10	1.46	0.54–3.94	25/63 (39.7)	2.86	1.55–5.28	2.62	1.33–5.16

*HB-HTS* home-based HIV testing and services

^a^Crude model includes adjustment for i.community and ii.uptake of HBHTC (for HIV-)/initiation timely ART (for HIV +) and iii. age as a priori confounding factors

Multivariable model for HIV- women additionally includes adjustment for the following: iv. marital status, v. employment status; vi. nights spent away from community; vii. control of household finances, viii. lifetime number of partners; ix. risk of alcohol dependence

Multivariable model for HIV + women additionally includes adjustment for the following: iv. nights spent away from community; v. control of household finances, vi. pregnancy in last 12 months; vii. risk of alcohol dependence; viii. risk of depression

^b^Too few values

Among women who reported having sex in the last 12 months (N = 216 HIV negative and N = 322 HIV positive), the most recent partner was most often reported to be the woman’s husband and the distribution of age difference with the most recent partner was spread across the age difference categories as shown in Table 2. A much larger proportion of HIV positive women reported condom use (59.6%, n = 192) than HIV negative women (15.7%, n = 34). Almost eight percent (n = 17) of HIV negative women and 13.0% (n = 42) of HIV positive women who reported having sex in the last 12 months disclosed alcohol use at the time of most recent sexual encounter. Approximately 9 10% of both HIV negative (n = 20) and HIV positive (n = 33) women reported transactional sex with the last sexual partner. Most HIV negative women who declared having sex in the last 12 months reported that the partner was not HIV positive to their knowledge (75.9%, n = 164) while 47.5% (n = 153) of HIV positive women reported that the partner was concordant HIV positive.

**Table 2:** Association of most recent sexual partnership characteristics with IPV, among those who reported sex in last 12 months, by HIV-status, among those who reported sex in last 12 months, by HIV-status

	HIV− [N = 216 median age:30y (IQR 23–38)]					HIV + [N = 322, median age 33y (IQR 27–39)]				
	IPV n/N (%)	OR^a^	95% CI	aOR^a^	95% CI	IPV n/N (%)	OR^a^	95% CI	aOR^a^	95% CI
Relationship to participant										
Husband	49/161 (30.4)	1	*p* = *0.30*	1	*p* = *0.76*	56/207 (27.1)	1	*p* = *0.75*	1	*p* = *0.75*
Boyfriend	11/54 (20.3)	0.62	0.25–1.54	0.76	0.13–4.53	32/108 (29.6)	0.91	0.49–1.66	0.96	0.43–2.16
Casual/one-off/other	0/1 (0)	b	b			1/7 (14.3)	b	b	b	b
Age difference with sexual partner										
Younger – ≤ 3y older	22/74 (29.7)	1	*p* = *0.44*	1	*p* = *0.66*	41/119 (34.5)	1	*p* = *0.03*	1	*p* = *0.15*
4 – 6y older	16/67 (23.9)	0.57	0.24–1.36	0.64	0.23–1.82	17/79 (21.5)	0.45	0.22–0.91	0.55	0.25–1.19
≥ 7y older	22/75 (29.3)	0.73	0.33–1.62	0.68	0.25–1.87	26/118 (22.0)	0.52	0.28–0.95	0.55	0.29–1.07
Condom use at last sex										
N	50/182 (27.5)	1	*p* = *0.67*	1	*p* = *0.30*	45/130 (34.6)	1	*p* = *0.03*	1	*p* = *0.07*
Y	10/34 (29.4)	1.22	0.49–3.07	1.84	0.58–5.83	44/192 (22.9)	0.56	0.33–0.94	0.58	0.33–1.04
Alcohol use at last sex										
N	52/198 (26.3)	1	*p* = *0.23*	1	*p* = *0.28*	70/280 (25.0)	1	*p* = *0.02*	1	0.14
Y	8/17 (47.1)	2.03	0.64–6.43	2.23	0.52–9.54	19/42 (45.2)	2.32	1.16–4.63	1.87	0.82–4.28
Transactional sex										
N	54/280 (19.3)	1	*p* = *0.02*	1	*p* = *0.05*	90/389 (23.1)	1	*p* = *0.99*	1	*p* = *0.99*
Y	10/20 (50.0)	3.63	1.28–10.30	3.97	1.02–15.37	8/33 (24.2)	0.99	0.41–2.38	0.99	0.36–2.75
Known/suspect HIV + partner										
N	37/164 (22.6)	1	*p* = *0.01*	1	*p* = *0.02*	17/69 (24.6)	1	*p* = *0.33*	1	*p* = *0.36*
Don’t know	21/44 (47.7)	2.75	1.22–6.16	3.01	1.24–7.29	24/100 (24.0)	1.03	0.49–2.14	1.25	0.54–2.92
Y	2/4 (50.0)	–^b^	–^b^	–^b^	–^b^	48/153 (31.4)	1.50	0.77–2.93	1.67	0.80–3.49

Multivariable model for HIV- women additionally includes adjustment for the following: iv. marital status, v. employment status; vi. nights spent away from community; vii. control of household finances, viii. lifetime number of partners; ix. Risk of alcohol dependence; x. transactional sex; xi. knowledge of partner’s HIV status

Multivariable model for HIV + women additionally includes adjustment for the following: iv. nights spent away from community; v. control of household finances, vi. pregnancy in last 12 months; vii. risk of alcohol dependence; viii. risk of depression; ix. age difference with partner; x. condom use at last sex; xi. alcohol use at last sex

^a^Crude model includes adjustment for i.community and ii.uptake of HBHTC (for HIV-)/initiation timely ART (for HIV +) to account for sampling strategy and iii. age as an a priori confounding factor

^b^Too few values

### Factors Associated with IPV

In a crude analysis of the association of age category with IPV in the last 12 months, there was weak evidence to suggest that older age was negatively associated with IPV among both HIV negative (p = 0.07) and HIV positive women; however, this relationship was not apparent in the multivariable analysis (p = 0.31 in HIV negative and p = 0.46 in HIV positive women) (Table 1). Among HIV negative women marital status was a strong predictor of IPV in the crude analysis (p < 0.001) and while evidence of the association weakened in the multivariable analysis (p = 0.07), the odds of IPV appeared lowest among “never married” women (aOR 0.26, 95% CI 0.08 0.87) but was also lower among “previously married” women (aOR 0.75, 95% CI 0.24 2.28) when compared to “currently married” women. There was weak evidence to suggest that employed HIV negative women were less likely to report IPV (aOR 0.43, 95% CI 0.17 1.07). Among HIV positive women, reporting autonomy over household spending was associated with 59% reduction in IPV (aOR 0.41, 95% CI 0.23 0.75) when compared to having no control over household finances. Reporting a higher number of lifetime sexual partners was associated with IPV among HIV negative women in the crude analysis (p = 0.006) but the association was not apparent in the multi variable analysis (aOR 2.06, 95% CI 0.89 4.77). Spending one or more nights away from the community in the last 12 months was associated with higher odds of IPV among both HIV negative (aOR 3.17, 95% CI 1.02 9.81) and HIV positive women (aOR:1.79, 95%CI:0.99 3.24). Pregnancy in the last 12 months (aOR 2.25, 95% CI 1.07 4.74), risk of alcohol dependence (aOR:2.75, 95%CI:1.51 5.00) and risk of mental distress (aOR 2.62, 95% CI 1.33 5.16) were all associated with increased odds of IPV among HIV positive but not among HIV negative women.

### Factors Related to Most Recent Sexual Partnership and Association with IPV in Women who Reported Sex in the Last 12 Months

Among HIV negative women who reported having sex in the last 12 months, those who reported transactional sex were almost four times more likely to report IPV in the last 12 months (aOR 3.97, 95% CI 1.02 15.07) and those who responded “don’t know” when asked if they knew or suspected their partner was HIV positive were more likely to report IPV (aOR 3.01, 95% CI 1.24 7.29). Among HIV positive women who reported having sex in the last 12 months, in the crude analysis women who had older partners were more likely to report IPV in the last 12 months (p = 0.03) and alcohol use related to most recent sex was associated with IPV (p = 0.03) but the associations were no longer apparent in the multivariable models (Table 2). There was some evidence that women who reported condom use during the most recent sexual partnership were less likely to report IPV but confidence intervals included 1 (aOR 0.58, 95% CI 0.33 1.04).

## Discussion

Approximately 1 in 5 women in our study from the HPTN 071 (PopART) communities in urban Zambia and South Africa reported experiencing sexual and/or physical violence, in the previous 12 months. This is consistent with other reports on the high prevalence of IPV in similar sub-Saharan African settings [19]. Few studies have examined factors associated with IPV by HIV status among women sampled from the same communities. We found similarities and differences in factors associated with IPV by HIV status.

While socioeconomic status was not obviously associated with IPV in the last 12 months, in both HIV negative and HIV positive women, those who had spent at least one night away from the community in the last 12 months (60%) were more likely to report IPV. Women who had travelled were not more likely to report being employed (including self-employed and informal employment), head of household or engaging in transactional sex. Other literature indicates that experience of IPV can precipitate travel, e.g. to return to a family home or village of origin to seek assistance from their families, or because their violent partner made them leave [20, 21]. Alternatively, or as well, IPV has been linked to short term migration and a loss of social networks or accusations of multiple partners [22].

Irrespective of HIV status, women who work were more likely to report financial autonomy although 58% (n = 57) of working HIV negative women and 46% (n = 70) of working HIV positive women still reported that they did not have control over household finances. We found that control of household finances was inversely associated with IPV. Greater financial independence has been shown to give women greater autonomy in relationships and this may be an underlying protective factor against IPV [23]. Successful interventions, such as the IMAGE intervention that combined micro-finance loans with a gender-empowerment training to increase financial autonomy of women, have seen reductions of IPV even 10 years after scale up of the programme [24].

The high prevalence of harmful alcohol use (16–20%) and mental distress (15–18%) among women in our study is concerning and also reflects strong associations which have been seen with IPV in other studies [9]. Among HIV positive women, these factors were both also associated with almost three times higher odds of reporting IPV. Among HIV positive (but not HIV negative) women, harmful alcohol use was also associated with mental distress. While dis-entangling cause and effect from the available data is impossible, the findings highlight the vulnerability of women experiencing these multiple factors simultaneously [9].Our findings also signal alcohol use as a concern which warrants intervention. DHS data from Zambia indicate that alcohol excess is associated with IPV, with women who reported that their partner is often drunk being much more likely to report IPV (84%) than women whose partner is sometimes drunk (59%) or does not drink alcohol (34%) [25]. Whilst there is less alcohol abuse reported in women than men in Zambia, increases in alcohol abuse and binge drinking are notable [26].

A recent study in South Africa identified the elevated risk of acquiring HIV through transactional sex [27]. In our study HIV negative women who reported transactional sex (among women who reported having sex in the last 12 months), were also four times more likely to report IPV, a finding that is supported by the literature that argues that transactional sex exists in a broader continuum of men's exercise of gendered power and control that is conducive to IPV [27, 28]. HIV negative women who were not aware whether their most recent partner may be HIV positive were also more likely to report IPV than women who said that their partners were not known or suspected to be living with HIV. This may suggest that among HIV negative women, those who were in less stable or trusting relationships were more likely to report IPV or that IPV could have led to more unstable partnerships. However, this was not the case among HIV positive women.

We must interpret all our observational findings with caution as women who experienced IPV may have been less likely to engage with research and report it. Women’s reports of violence and associated factors must be interpreted in light of reporting biases encountered with all self-reported data, namely social desirability and recall bias. Because of the broad scope of the studies which collected the data involved in this analysis, only two questions were used to enquire about IPV and this is likely to have reduced reporting [29]. Other limitations of our data include the fact that approximately 40% of HIV negative women (n = 135) self-reported their HIV negative status, having declined HIV testing. Some HIV positive women also self-reported HIV status but verification of HIV positive status was usually sought and it is probably safe to assume that very few participants falsely report living with HIV. When data on HIV negative women are restricted to those who accepted HB-HTS so that HIV status could be verified, one meaningful difference was noted. Among the restricted group, nights spent away from the community showed no indication of association with IPV in the last 12 months in contrast to the wider group which included self-reported HIV negative women who were three times more likely to experience IPV if they had been mobile. The study population consisted of individuals recruited for two separate earlier studies and was stratified by uptake of HB-HTS (among HIV negative women) and timely initiation of ART (among HIV positive women) and this has to be borne in mind when interpreting our results although there is no evidence for a resultant differential selection bias in relation to IPV. The two separate samples are also the reason we did not conduct tests for interaction by HIV status. We accounted for community in our analyses but the study was not powered to identify differences between the two countries involved.

The prevalence of IPV was not associated with uptake of home-based testing or timely ART initiation among women who participated in our study suggesting that women experiencing IPV were not excluded by PopART interventions. This is encouraging for ensuring universal coverage with similar approaches and may be a consequence of specific training in IPV support being provided for community-health workers delivering the intervention. Existing data on HIV and experience of IPV are conflicting. Our study sheds light on factors associated with IPV by HIV status in women living in urban sub-Saharan Africa. Our findings that HIV positive women who report IPV are also affected by potentially modifiable co-morbidities namely harmful alcohol use and mental distress, are worthy of urgent further attention. Our finding that mobility as defined by nights spent away from the community in the last 12 months was associated with IPV in the last 12 months among both HIV negative and HIV positive women should be a particular area of further research as mobile populations are also more at risk of HIV, STIs and other risks to health [30]. Qualitative methods to tease out more nuanced aspects would add to current knowledge. For instance, migration may take on many forms (in terms of frequency, duration, regularity, single or multiple destinations etc.) and the reasons for it could be manifold. While our data shed light on mobility as a factor associated with IPV, other research methods could build on these quantitative findings to understand more about IPV in these settings. In addition, better understanding of approaches to improve women’s options for protecting themselves, including active non-governmental organisations or state institutions which women can access would be helpful. The What Works to Prevent Violence against Women and Girls programme has shown numerous interventions that prevent and address IPV among different populations and a recent systematic review highlighted those relevant for young people affected by HIV, including relationship-level interventions, microfinance combined with gender-transformative approaches such as IMAGE and community mobilisation interventions to change social norms [31, 32].

In conclusion, self-report of IPV was common in the HPTN 071 (PopART) study communities and our study has highlighted several important areas for the attention of health providers, policy makers and researchers.
